# The response of sea turtles to vocalizations opens new perspectives to reduce their bycatch

**DOI:** 10.1038/s41598-024-67501-z

**Published:** 2024-07-17

**Authors:** Damien Chevallier, Léo Maucourt, Isabelle Charrier, Pierre Lelong, Yves Le Gall, Eric Menut, Bryan Wallace, Cyrielle Delvenne, Orsolya Vincze, Lorène Jeantet, Marc Girondot, Jordan Martin, Ouvéa Bourgeois, Muriel Lepori, Pascal Fournier, Christine Fournier-Chambrillon, Sidney Régis, Nicolas Lecerf, Fabien Lefebvre, Nathalie Aubert, Mosiah Arthus, Matthieu Pujol, Michel Anthony Nalovic, Moulanier Nicolas, Marie-Clémence Burg, Pascale Chevallier, Tao Chevallier, Antony Landreau, Stéphane Meslier, Eugène Larcher, Yvon Le Maho

**Affiliations:** 1 BOREA Research Unit, Laboratoire de Biologie des Organismes et des Ecosystèmes Aquatiques, MNHN, CNRS 8067, SU, IRD 207, UCN, UA, Station de Recherche Marine de Martinique, Quartier Degras, Petite Anse, 97217 Les Anses d’Arlet, France; 2 Université des Antilles, Campus de Schoelcher, 97275 Schoelcher Cedex, France; 3 Institut des Neurosciences Paris-Saclay, CNRS, Université Paris-Saclay, 91400 Saclay, France; 4 Ifremer, Service Acoustique Sous-marine et Traitement de l’Information, Direction de la Flotte Océanographique, ZI de la Pointe du Diable - CS 10070, 29280 Plouzane, France; 5 Ecolibrium, Inc., 5343 Aztec Drive, Boulder, CO 80303 USA; 6 Department of Ecology and Evolutionary Biology, University of Colorado, 1900 Pleasant St, Boulder, CO 80302 USA; 7 LIttoral, Environnement et Sociétés (LIENSs), UMR 7266, CNRS Université de La Rochelle, 2 rue Olympe de Gouges, 17042 La Rochelle Cedex, France; 8 African Institute for Mathematical Sciences, 7 Melrose Rd, Muizenberg, Cape Town, 7950 South Africa; 9 Department of Mathematical Sciences, Stellenbosch University, Victoria Street, Stellenbosch, 7602 South Africa; 10 CNRS, AgroParisTech, Ecologie Systématique et Evolution, Université Paris-Saclay, 91190 Gif-sur-Yvette, France; 11 Groupe de Recherche et d’Etude pour la Gestion de l’Environnement, Route de Préchac, 33730 Villandraut, France; 12 Association ACWAA, Quartier l’Etang, 97217 Les Anses d’Arlet, France; 13 Solda Lanmè - Caribbean Sea Soldier, 61 rue Anca Bertrand, Cité Dillon, 97200 Fort de France, France; 14 Fishingcleaner.com, 78 Rue Justin Catayee, 97300 Cayenne, France; 15 ANSLO-S Association naturaliste de soutien logistique à la science, 7 Avenue Georges Clémenceau, 49280 La Tessoualle, France; 16 Mairie des Anses d’Arlet, Boulevard des Arlésiens, 97217 Les Anses-d’Arlet, France; 17 Université de Strasbourg, CNRS, IPHC UMR 7178, 23 rue Becquerel, 67000 Strasbourg, France

**Keywords:** Behavioural methods, Ecology, Ecology

## Abstract

Incidental capture of non-target species poses a pervasive threat to many marine species, with sometimes devastating consequences for both fisheries and conservation efforts. Because of the well-known importance of vocalizations in cetaceans, acoustic deterrents have been extensively used for these species. In contrast, acoustic communication for sea turtles has been considered negligible, and this question has been largely unexplored. Addressing this challenge therefore requires a comprehensive understanding of sea turtles’ responses to sensory signals. In this study, we scrutinized the avenue of auditory cues, specifically the natural sounds produced by green turtles (*Chelonia mydas)* in Martinique, as a potential tool to reduce bycatch. We recorded 10 sounds produced by green turtles and identified those that appear to correspond to alerts, flight or social contact between individuals. Subsequently, these turtle sounds—as well synthetic and natural (earthquake) sounds—were presented to turtles in known foraging areas to assess the behavioral response of green turtles to these sounds. Our data highlighted that the playback of sounds produced by sea turtles was associated with alert or increased the vigilance of individuals. This therefore suggests novel opportunities for using sea turtle sounds to deter them from fishing gear or other potentially harmful areas, and highlights the potential of our research to improve sea turtles populations’ conservation.

## Introduction

**Figure 1:**
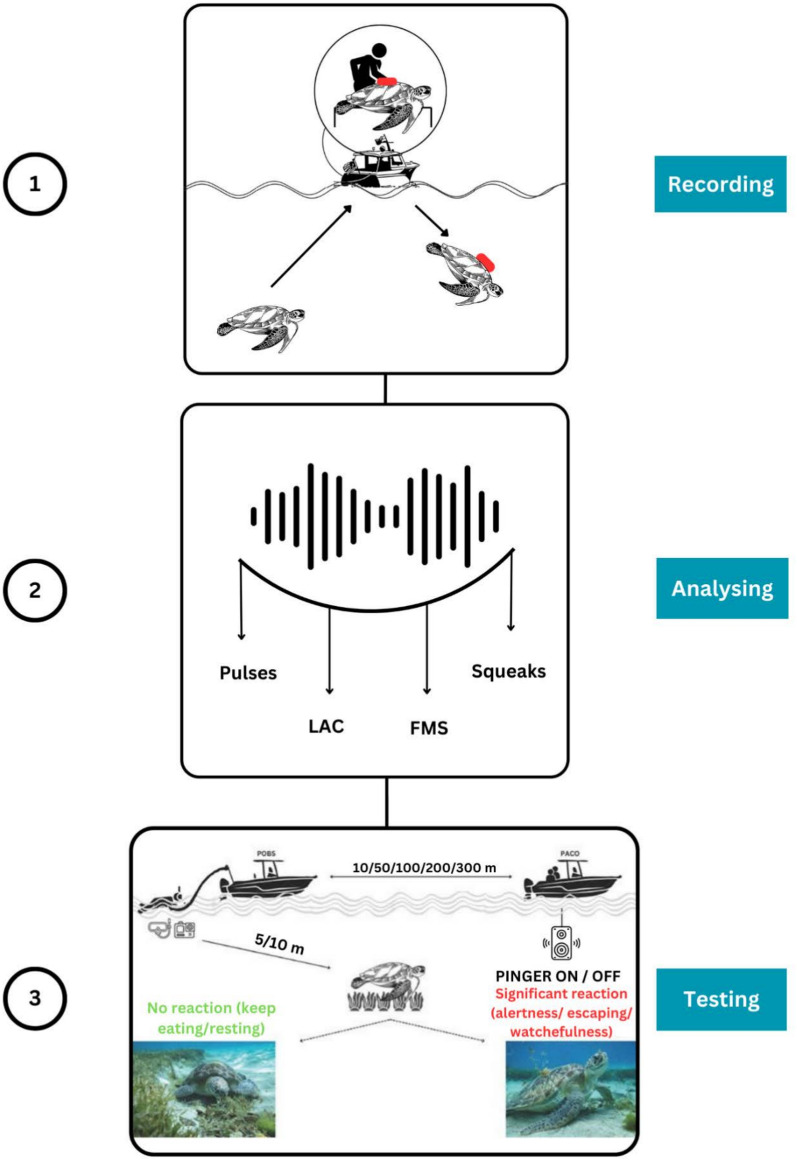
Schematic illustration of playback tests (POBS: observation platform; PACO: acoustic platform) and illustration of immediate response types (0 = no reaction; 1 = significant reaction).

Sea turtle bycatch, a major threat to many species, occurs in industrial and artisanal fisheries using a variety of gear types including longlines; gill nets; trawls; traps; and pots^1–5^. Bycatch threatens sea turtles globally since the areas where fisheries operate overlap with sea turtle foraging habitats, breeding grounds and migratory corridors both spatially and temporally in coastal and offshore ocean areas. In the French West Indies, fishing holds immense economic significance, estimated at 20 M€/year. The predominant artisanal fishing practices involve small, single-person fishing companies, utilizing vessels under ten meters in length. These operations encompass coastal operations, focused on demersal resources, and offshore operations targeting pelagic species (Scombridae, Istiophoridae, Coryphaenidae, etc.). Coastal fishing constituted 62% of active vessels in Martinique in 2019 and 65% in Guadeloupe in 2018. Various types of nets targeting different species (e.g. conch, lobster, reef fish) are used, including trammel nets and entangling gillnets set at the surface or ocean bottom. Although sea turtles are known to interact with all of these (and other) fishing gears, characterizing these interactions remains challenging. The prohibition of sea turtle fishing in Guadeloupe (1991) and Martinique (1993) has somewhat contributed to the preservation of sea turtle populations, but accidental captures of sea turtles persist. Bycatch represents a significant threat and risk of direct mortality for juvenile and adult green turtles frequenting coastal waters in these territories. A single study^6^ carried out in Martinique on the impact of its gillnet fishery, reports that more than 800 green sea turtles (*Chelonia mydas*) are unintentionally captured annually. Though this study dates back to almost a decade it illustrates the potential threat coming from this fishery’s bycatch. For fishermen, these captures not only result in diminished earnings due to the reduced catch of target species but also incur additional costs (expenses and time) for repairing or replacing damaged gear. The complexity of this situation makes effective communication challenging, but requires strong collaboration with fishermen willing to contribute to finding solutions. The impact of bycatch on coastal fisheries management is substantial, sometimes leading to the closure of fisheries. Consequently, there is an urgent need to develop technologies to reduce bycatch, especially that of sea turtles. This is essential in order to protect sea turtles while also securing the livelihoods of local fisheries^7^. Existing literature highlights diverse techniques designed to contribute to the reduction of sea turtle bycatch in gillnets, while also maintaining an acceptable fishing yield^8–13^. The development of these technologies relies on differences in sensory systems of sea turtles and those of target species of fisheries. The use of visual deterring devices (Visual Deterrent Devices, VDD), particularly green and UV LEDs, appears to reduce turtle bycatch in some fisheries. However, understanding of the specific behavioral responses of turtles to these stimuli remains limited; i.e. whether illuminating gear alerts animals of its presence to avoid physical interactions or scares them away from the gear^14^. Despite apparent success in reducing sea turtle bycatch, net illumination remains largely experimental, and has not been implemented at scale in commercial fisheries for turtle bycatch reduction purposes. The application of these devices presents challenges for fishers ranging from entanglement in nets, concerns over the durability of LEDs, and the associated financial burden of acquiring and maintaining them. Furthermore, the primary batteries employed in these devices are Li-ion batteries, raising environmental concerns due to disposal of spent batteries and water-intensive lithium extraction, resulting in issues such as soil pollution and the depletion of water reserves^15–17^. In this context, experiments designed to evaluate the impact of low frequency Acoustic Deterrent Devices (ADDs) on sea turtle behavior might reveal a more efficient alternative solution to sea turtle bycatch reduction. Behavioral and electrophysiological studies explored the acoustic ecology of sea turtles, focusing on their auditory capabilities, their responses to acoustic stimuli and the implications of this knowledge for their conservation^7,18–21^. Their research measured the underwater hearing sensitivities of juvenile green, juvenile and adult loggerhead, hatchling leatherback (*Dermochelys coriacea*), and hatchling hawksbill sea turtles by recording potential responses to synthetic tonal stimuli. They concluded that sea turtles are able to perceive sound signals in a range from 50 to 1600 Hz, with a maximum sensitivity between 10 and 400 Hz^7,18,21^. In addition, though sea turtles have long been believed to be silent, recent studies identified sound production in hatchling^22–25^ and in juvenile green sea turtles^3^. Our primary objective in the present study was therefore to explore whether turtle sound production, especially those associated with alertness, escape behavior, or social contact, could provide a suitable tool for mitigating turtle bycatch. To accomplish this objective, we explored variation in behavioral responses of foraging sea turtles to synthetic sound signals and natural sounds produced by green turtles (“Online Methods” and Fig. 1).

**Figure 2:**
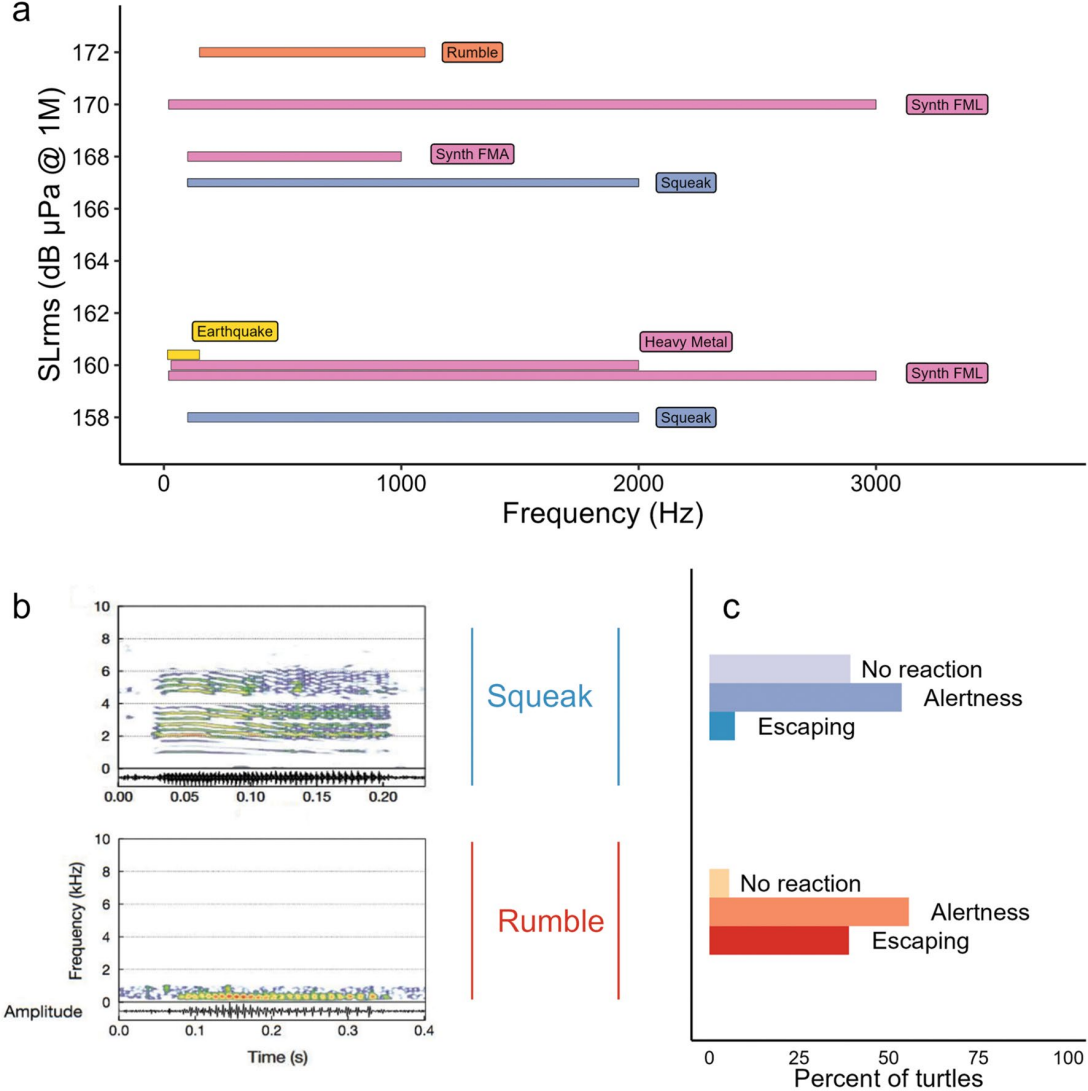
(**a**) Frequency (Hz) and sound level (mean dB µPa @1 m rms) of the presented signals tested during the study (Synthetic sounds are represented by pink rectangles), (**b**) Spectrograms of Squeak (top) and Rumble (bottom) recorded from wild green turtles^21^ and (**c**) percent of turtles for each type of reaction to these two signals (respectively: Squeak in blue shades and Rumble in orange shades).

In a first step, we recorded the sounds produced by free-ranging juvenile green turtles and their behaviors using on-board camera devices and hydrophones attached to their carapace in Martinique (detailed methodology described in Refs.^26,27^. Overall, we recorded and described 10 sounds produced by green turtles and we identified four main sound categories for sounds produced: pulses, low amplitude calls (LAC), frequency modulation sounds (FMS), and Squeaks^26^. In a second step, we examined the behavioral responses of green sea turtles foraging in their natural environment to sounds which could potentially be associated with fear, flight or social contact: Rumble (LAC category) and Squeak (Fig. 2a). The lack of knowledge about the behavioral reactions of marine turtles to sound waves (exact range of frequencies, nature of the signals: impulsive or not, synthetic or natural, etc.) led us to develop two acoustic sources with different characteristics. These are described in more detail in the Online Methods section. The first acoustic source (electrodynamic class) is suitable for very low frequency (from 20 Hz) and broadband (up to 3 kHz) modulated signals.

The second acoustic source (piezoelectric class) has a narrower bandwidth and is poorly suited to frequencies below 150 Hz and above 1 kHz. However, it is capable of reproducing impulse sounds with a higher efficiency than the first source. The Synth FML, Synth FMA and Rumble signals were mainly produced by the piezoelectric source. Heavy metal playbacks and Earthquake sounds were only transmitted from the electrodynamic source. Squeak-type signals were tested using both acoustic sources.

Five different recordings of the Squeak signal were presented to the turtles, varying in frequency, duration or intensity (see details in “Table 2”). These five recordings were presented as a single acoustic signal in the tests. A geophonic sound (Earthquake) and three synthetic sound signals (Synth FML, Synth FMA and Heavy Metal playback) were additionally tested (Fig. 2a). We used two small vessels to broadcast signals and observe behavioral responses. One vessel, referred to as the "observation platform" (POBS) was employed by a diver responsible for spotting and locating turtles underwater. Upon spotting an individual, the POBS informed the second vessel, equipped with the acoustic platform (PACO). The PACO then positioned itself in proximity of the observed turtle as the diver looked on and activated the speaker and initiated sound playback. The POBS’s diver observed and recorded (using a GoPro Hero 10 device) the behavior of the target individual (Fig. 1). Visual observations were quantified using two metrics: (i) assessing the immediate impact of sound playback on the behavior of green turtles (referred to as “shot” hereafter), with reaction intensity rated on a scale of 0 (no reaction), 1 (significant reaction with alertness or watchfulness, i.e. turtle raises suddenly its head and look around, Fig. 1), to 2 (escaping, i.e*.* turtle swimming rapidly away from the test area); Fig. 1) and (ii) assessing the change in activity by comparing the behavior recorded before and after each shot. Several trials were performed, each one involved the repetition of shots of a given signal on an individual turtle at variable distance (5–250 m), and the PACO moved then closer to the animal, but always remained at a distance greater than five meters. Two alternative versions of this protocol were used (1) to determine which sound signal triggered the turtle behavioral responses and (2) to measure the distance and habituation effect to this sound. For the first aim, trials are stopped if the turtle escapes and are composed of up to three shots. We then tested the immediate reaction of a given individual to a defined signal within a trial. For the second aim, only sounds that elicited the highest number of behavioral responses were tested with up to 13 shots per trial, on a wide range of distances (40–500 m).

A total number of 75 initial trials to assess turtle response to each tested sound were performed on 68 individuals to assess the reaction of turtles to the different signals, with an average of 2.63 ± 0.65 shots per trial. Secondly, 20 trials on 20 individuals were carried out to test the distance and habituation effect of particular sounds that elicited the highest level of behavioral responses, involving a mean of 5.40 ± 2.76 shots per trial.

### Reaction to signals

The three *synthetic sound signals* were also tested in 17 (Synth FML), five (Synth FMA) and three (Heavy Metal playback) trials performed on 23 different feeding turtles. We observed no reaction to any of these synthetic sounds. The geophonic sound (Earthquake) was tested on four feeding turtles, triggering no reaction in any of the tested individuals. We presented the natural sounds produced by sea turtles, the Rumble (Fig. 2b) and the Squeak (Fig. 2b), in playback tests to 18 and 28 feeding turtles, respectively. Probability of reaction to sea turtle sounds (all confounded) was estimated at 0.95 (Credible interval 95% (CI_95%_) 0.74 − 1.00) while reaction to Earthquake and Synthetic signals are estimated at 0.00 (CI_95%_ 0.00 − 0.0001) and 0.00 (CI_95%_ 0.00 − 0.00005) respectively. There was then a significant difference in the proportion of turtles reacting to the sounds produced by sea turtles, with 17 of total 18 turtles (94.4%) reacting to the Rumble and 17 of total 28 (60.7%) to the Squeak by exhibiting either a vigilance posture, escaping, or a combination of the two. More precisely, Rumbles triggered only vigilance in 55.6% of observed responses, immediate escape or vigilance followed by an escape in 38.9% of observations, and triggered no reaction in 5.6% of the tested individuals (Fig. 2c). Squeaks triggered vigilance in 53.6%, immediate escape or vigilance followed by an escape in 7.1%, and no reaction in 39.3% of the tested turtles (Fig. 2c). The proportions of “No Reaction” and “Escaping” behaviors varied significantly between the broadcast of Rumbles and Squeaks with a higher probability of escape behavior for Rumble (median difference of 0.32, CI_95%_ 0.09 − 0.56) and higher frequency of no reactions for Squeak (median difference of 0.34, CI_95%_ 0.13 − 0.55). Nonetheless, alertness reaction proportions did not differ significantly between the two signals.

### Distance and habituation effect

**Figure 3:**
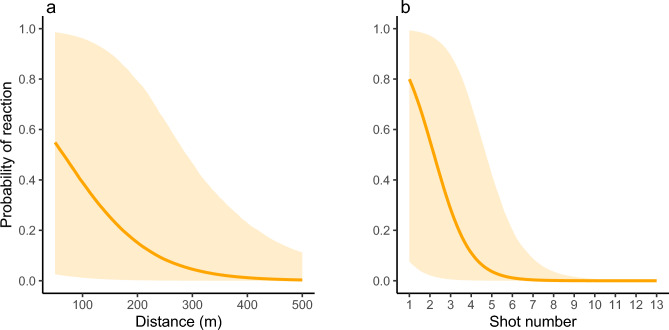
Probability of response to Rumbles with 95% credible interval obtained from Bayesian generalized linear mixed model according to distance (m) (**a**) and shot number (**b**).

Shots were played at different distances using the Rumble signal. When the Rumble signal was played from a distance of > 300 m from the target individual (n = 17 shots), all shots resulted in no reaction. When the playback tests were performed from a distance between 200 and 300 m (n = 23 shots), 26.1% of turtles changed their behavior, 45.9% changed their behavior from a distance between 100 and 200 m (n = 37) and 38.7% from a distance < 100 m (n = 38 shots) from the focal individual. The distance between the focal turtle and the source of sound had thus a significant effect on the likelihood of turtles to react, with an increasing probability of reaction when this distance decreased (CI_95%_ − 0.03 − − 0.01, Fig. 3a). Turtle’s reactions occurred mainly for the first, the second and the third shots with 70% (n = 20), 60% (n = 20) and 29.4% (n = 17) of reactions respectively, regardless of the distance at which the shots were played. Turtles seemed to react less frequently after the fourth, fifth and sixth shots with 6.2% (n = 16), 18.2% (n = 11) and 12.5% (n = 8) of reactions respectively. Beyond six shots, turtles stopped reacting to Rumble signal. The probability of turtles’ responsiveness was inversely related to the number of shots (CI_95%_ − 1.85 − − 0.71, Fig. 3b).

The use of acoustic deterrents (pingers) for cetacean bycatch reduction has been successful because of the reliance of these species on acoustics for their general ecology and life history. In contrast, there was a general assumption that acoustic communication is negligible in sea turtles. For our knowledge, our study then demonstrates for the first time that sea turtles behaviorally respond to the sounds they produce, and that their vocal repertoire is more functional than previously thought. These findings therefore open new possibilities to reduce bycatch since acoustic signals could be deployed with various fishing gears to potentially reduce sea turtle interactions. Pingers were first developed in the late 1990s to keep porpoises away from U.S. fish farms. Today there are around ten different models (banana or cylinder shaped), which can be installed on fishing nets^28^. Once the sounds produced by sea turtles are identified, the principle would be to emit sounds in the frequency ranges of green turtles (between 200 and 400 Hz), to raise their awareness of a potential threat and to trigger them to move away from the area where the fishing equipment is deployed. This approach, if successful, could also be applied in other subaquatic equipment that are potentially dangerous to sea turtles (e.g. boat propellers, dredging equipment, etc.). The development and eventual implementation of such bycatch reduction technologies could be eligible for significant financial support from the French and European governments. As an example from 2023 the gillnet fleet in the Bay of Biscay on the Atlantic coast of France obtained a financial envelope of 6 million euros, which represents 100% of the funding requested to equip its boats with pingers in order to reduce the accidental captures of common dolphins. The publicly funded project covered 100% for experimental device costs, and 50% for devices already considered to be marketable. Further, there is potential applicability of this approach to other marine turtle species and other taxons.

However, as the effectiveness of acoustic methods likely varies depending on the species and its life stage, acoustic studies need to be carried out around the world to record the sounds of different species and different populations of sea turtles. Thus, targeted studies on the recording, analysis and cataloging of the sounds of different species of sea turtles would increase the likelihood of improving the effectiveness of acoustic methods and approaches. This should be a research priority, particularly for researchers working on fine-scale underwater turtle behavior and bycatch mitigation measures.

Despite the intensive investigation of many aspects of sea turtle life-history in the wild for over four decades, their underwater communication capacities and behavioral responses to sound have gone largely overlooked. Our recent findings about sounds produced by sea turtles in the French West Indies island of Martinique elicit strong interest and therefore present new opportunities to reduce harmful interactions between turtles and fishing gear.

### Supplementary Information

Supplementary Information.

## Data Availability

The data that support the findings of this study are available from Damien CHEVALLIER but restrictions apply to the availability of these data, which were used under license for the current study, and so are not publicly available. Data are however available from Damien CHEVALLIER (damien.chevallier@cnrs.fr) upon reasonable request and with permission of Damien CHEVALLIER.
