# Supplementary material for: The response of sea turtles to vocalizations opens new perspectives to reduce their bycatch
Source: Sci Rep. 2024 Jul 17;14:16519. doi: 10.1038/s41598-024-67501-z (PMC11255315; doi:10.1038/s41598-024-67501-z)
Supplement: Supplementary file 1 — Supplementary Information. [file 41598_2024_67501_MOESM1_ESM.docx]

**ONLINE METHODS**

Methods and any associated references are available in the online version of the paper.

**Study area and permits.** This study was carried out March 2023 in Anses d’Arlet (14° 50’ N, 61° 9’ W), Martinique Island (French West Indies, France). We conducted our study in five bays of Les Anses d′Arlet (14◦30′ 9.64′′ N, 61◦5′11.85′′ W): Anse Noire, Anse Dufour, Grande Anse, Anse du Bourg and Anse Chaudière. The intentional disturbance of green sea turtles met French ethical and legal requirements. The CNRS protocol was indeed approved by the “Conseil National de la Protection de la Nature”and the “Ministère français de l'Écologie, du Développement Durable et de l’Energie (permit number: 971-2022-11-24-00004). The fieldwork was carried out under the certification of Damien Chevallier (prefectural authorizations’ owner) under strict compliance of the Policy of Martinique’s recommendations in order to minimize animal disruption.

**Green sea turtle sound production.** We first characterized the sounds produced by green sea turtles and identified the associated behaviors by deploying on-board video cameras and hydrophones on n = 23 free-ranging green turtles in Martinique between 2018 and 2022. The device was attached to their carapace using four suction cups and was released via an automatic release system (Jeantet et al., 2020). The hydrophone recorded in mono at a sampling rate of 24 kHz (16 bit). The audio recordings were analyzed, and the green turtle sounds were manually identified by listening to and visualizing the spectrograms. The vocal repertoire of green turtles has previously been described by Charrier et al. (2022). Each sound emitted by green turtle was extracted and categorized based on its duration, pulse rate, frequency, and Sound Pressure Level (SPL) into the four categories identified by Charrier et al.: Pulse, Low Amplitude Call (LAC), Frequency Modulation Sound (FMS), and Squeaks. The visualization of behaviors associated with the sounds emitted by the green turtles allowed us to identify LAC and Squeak as sound categories potentially linked to fear, flight, or social contact. Subsequently, the highest quality sound samples in the LAC and Squeak categories were selected, with the acoustic parameters for each signal indicated in Table 2. These sounds were then played using a speaker in the presence of green sea turtles foraging in their natural environment to examine their behavioral responses to these signals.

**Acoustic signals protocols.**

***Characteristics of the two transmission chains.*** Two autonomous very low frequency transmission chains with internal battery have been developed:

- A so-called electrodynamic chain consisting of a prototype electrodynamic loudspeaker resonating at 70 Hz and dedicated custom-made power supply and electronics. This chain can sweep the frequency band [20-500 Hz], with a maximum peak sound level (SL_pk_) of 169 dB (ref. 1 µPa @ 1 m). A broadband prototype speaker [20-3000 Hz] can also be used. In this case, the very low frequency energy ([20-500 Hz]) is attenuated compared to that obtained with the nominal loudspeaker (emission level reduced by approximately 5 dB in the VLF band).

- A so-called piezoelectric chain made up of a piezoelectric transducer resonating at 180 Hz (Geospectrum Bender M21-325-200) and specific custom-made power supply and electronics. This chain can sweep the frequency band [100-1000 Hz], with a maximum peak sound level (SL_pk_) of 173 dB (ref. 1 µPa @ 1 m). This piezoelectric source is less energetic in the band [20-150 Hz] than the electrodynamic source, but works up to 1 kHz, for an identical average emission level. The emission levels given in Table 2 come from measurements made from a hydrophone (OceanSonics IcListen HF) deployed from the PACO acoustic platform during the experiment.

***Signals tested.*** Table 2 summarizes the different sound signals used for the first playback tests. Different frequency modulations (FM) were tested on the green sea turtles: linear FM (FML), random FM (FMA) and summation of linear FM (NFML). Different frequency bands were scanned depending on the transmission chain: [20-500 Hz], [20-3000 Hz] and [100, 1000 Hz], with acoustic energies distributed differently in the band depending on the transmitter used. For these FMs, the maximum peak sound level (SL_pk_) was of the order of 173 dB (ref. 1 µPa at 1 m). This is a very moderate emission level.

In addition to these frequency modulations, the following were tested:

- A sound of the geophony, an earthquake (TT), covering the range 15-150 Hz, with an energy peak at 50 Hz. The emission level of this sound sequence was lower than that of the FM mode.

- Two sound types produced by sea turtles (Squeak or Rumble) and broadcast on one of the transmission channels (the choice of channel and HP depends on the frequency content of these signals).

***Physiological impact of the selected signals.*** Published literature on the physiological impact thresholds of sea turtles is notably scarce. However, the US Navy has proposed threshold values for sea turtles (Table 1^24^), in function of PTS (Permanent Threshold Shift) and TTS (Temporary Threshold Shift). In an animal, exposure to sufficiently intense sound can result in an elevation of hearing threshold. The duration of this threshold increase primarily hinges on factors such as exposure time, signal amplitude and its frequency. This change in hearing threshold can be either temporary (TTS) or permanent (PTS). To estimate the impact radius of an acoustic source, calculations are derived from these thresholds and the levels at the source (at the reference distance of 1 m). Two metrics, “peak level” and “cumulative sound exposure level”, have been used during this study. The cumulative sound exposure level (SEL_CUM_) integrates all the sound sequences received by the animal, taking into account transmission losses based on the distance between the sound source and the exposed turtle.


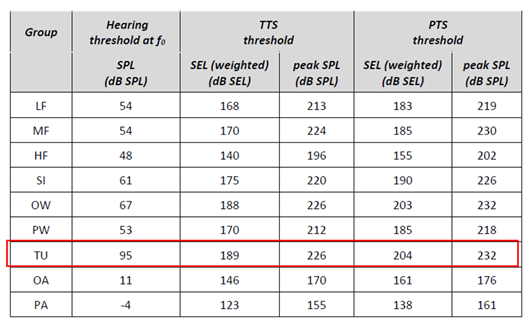


**Table 1** Sea turtle PTS and TTS thresholds (red box)^1^.

***Peak level impact received by the animal.*** The received peak level TTS threshold was therefore estimated to: 226 dB ref. 1µPa. Since sound levels (SL_pk_) were very moderate (maximum 175 dB ref. 1 µPa @ 1 m by taking 2 dB safety factor for the sound risk analysis), the threshold was never reached. There was therefore no predicted deleterious impact on sea turtles, even temporary, regardless of the distance.

***Impact in cumulative sound exposure level received by the animal.*** The TTS threshold in cumulative sound exposure level was therefore 189 dB ref. 1 µPa^2^.s. For one acoustic emission, the maximum sound exposure level (SEL_1shot_) was 178 dB ref. 1 µPa^2^.s at 1 m. For a turtle at 1 m from the acoustic source, the cumulative sound exposure level threshold was reached after 13 emissions. For a turtle at 5 m from the acoustic source, the cumulative noise exposure level threshold was reached after approximately 300 emissions. For a turtle at 10 m from the acoustic source, the cumulative sound exposure level threshold was reached after approximately 1300 emissions. In view of these data, we decided very cautiously not to use acoustic sources within five meters of sea turtles. The levels of peak and sound exposure implemented during these tests were low enough to guarantee that no physiological impact, even temporary, could be induced for the targeted species. Importantly, this study was interested in turtle reactions function of signal type (synthetic vs sound produced by turtles). Thus, the reaction of turtles induce by a level of signal emissions beyond threshold could bias the results, and should be avoided. In this study, we favor the particular structure of an alert signal over the amplitude of a repulsive signal.

**Acoustic Deterrent Device Behavioral Tracking and Trials.** The effectiveness tests of the signals used on the sea turtles were carried out using two light boats:

- A boat called "platform observation" (POBS) to tow an underwater observer responsible for spotting turtles underwater. Once a turtle had been spotted, the POBS checked that the turtle is feeding and informed the acoustic platform (PACO). The POBS underwater observer observed and recorded video (GoPro Hero 10) of any immediate changes in behavior (**Fig. 1**).

- A boat called "acoustic platform" (PACO) from which the sound sources were implemented. The loudspeaker was deployed as a pendulum at a depth of 1.5 (electrodynamic) to 5 meters (piezoelectric) providing that the water depth was sufficient. The autonomous electronics box was on board. The choice of the signal and its amplitude level were controlled from an onboard PC and a Bluetooth connection with the box. Different signals could be tested on the same animal. We have not grouped "Squeak" and "Rumble" into a single sound in order to avoid bias in the reactions. The test of a signal on an animal is called a “trial”, which is constituted of several “shots” corresponding to signal emissions. Each “shot” last between 3 and 12 seconds, depending of the signal (Table. 2). The interval between two shots was 12 seconds.

The visual observation of the turtles' reactions was carried out at 2 levels:

- Measurement of the immediate effect on behavior at the time of the "shot", with estimated intensity (0=no reaction, i.e. continue to feed; 1=alertness and 2=escaping) (**Fig. 1**)

-Analysis of the change in activity rhythm by comparison of the behavioral observation sequences before and after "shooting".

For each type of sound signal, the initial source-animal distance was maximum 250 meters and there was only one initial transmission. If the turtle did not escape, the test continued up to three identical shots. PACO boat would then come closer to the animal, however remaining at a distance greater than 5 m.

This method was then used a second time using a single signal, with a wider range of distance from 40 to 500m and up to 13 repetitions, in order to obtain dataset for distance and habituation effects testing. Taking into account the specific propagation conditions in very shallow waters, it is estimated that the SPL_rms_ (i.e. the sound level received by the turtles) from which behavioral reactions are observed on green turtles with "Rumble" type signals are of the order of 135 dB ref.1 µPa.

**Statistical analyses.**

In order to test the behavioral reaction to emitted signals, two datasets were used. The first dataset was built to determine the probability of response to the different categories of signal. A response in this sense could be alertness or escaping and are coded as “1” and “2” respectively (as opposed to no response observed coded as “0”). The first aim was to model the probability of response (alertness and escaping combined) function of the category of signal (Earthquake, Synthetic or Sea turtle sounds). Then, for the signals to which turtles responded the most, the number of turtles is counted for each type of possible response as well as for non-response. Inside a trial, only the strongest response behavior to a signal was retained (ordered by increasing strength: 0: “no reaction”, 1: “alertness”, 2: “escaping”). The second aim is thus to model the proportions of the different possible responses for each selected signal.

The second dataset, created only using the signal eliciting the strongest behavioral response, was built to model the distance and the habituation effect on turtle reaction. The turtle behavioral response variable is binomial (1: alertness or escaping, 0: no reaction) and was modeled accounting for distance of the shot to the turtle and shot number.

For each analysis, Bayesian Generalized Linear Mixed models (GLMMs) were set up with uninformative priors using ‘brms’^2^ and ‘coda’^3^ packages in R v4.2.2^4^. Trials ID were included as random effects to model the probability of response function of the category of signal and the distance and habituation effects. An effect is considered to be of potential interest when the credible interval 95% (CI_95%_) did not include 0. Two estimates are considered as potentially different when their CI_95%_ did not overlap.

| **Transmission chains** | **Track** | **Signals tested** | **Frequency band**  **(Hz)** | **Duration (s)** | **SPL_rms_  (dB µPa @ 1m)** | **Test date** (March 2023) | **Number**  **of trials** |
| --- | --- | --- | --- | --- | --- | --- | --- |
| **ELECTRO LB** | TRACK 7 | SQUEAK | 100-2000 | 4 | 158 | 14 | 3 |
|  | TRACK 10 | SQUEAK | 100-2000 | 12 | 158 | 14 | 5 |
|  | TRACK 9 | SQUEAK | 150-1100 | 3 | 158 | 14 | 3 |
|  | TRACK 4 | SYNTH FML | 20-3000 | 5 | 160 | 14 | 1 |
|  | TRACK 11 | SQUEAK | 100-2000 | 5 | 158 | 15-16 | 11 |
|  | TRACK 13 | HEAVY METAL | 30-2000 | 5 | 160 | 15 | 3 |
| **ELECTRO V5** | TRACK 1 | NAT TT | 15-150 | 11 | 160 | 16 | 4 |
| **PIEZO** | TRACK 3 | SYNTH FMA | 100-1000 | 5 | 168 | 16-17-21 | 4 |
|  | TRACK 1 | SYNTH FML | 100-1000 | 5 | 170 | 17-20 | 4 |
|  | TRACK 2 | SYNTH FML | 100-1000 | 7 | 170 | 16-17-20 | 12 |
|  | TRACK 4 | SYNTH FML | 100-1000 | 5 | 170 | 17 | 1 |
|  | TRACK 5 | RUMBLE | 150-1100 | 3 | 172 | 17-20-21-22 | 38 |
|  | TRACK 6 | SQUEAK | 100-1300 | 4 | 169 | 20-21 | 6 |
|  |  |  |  |  |  | **Total** | **95** |

**Table 2** Acoustic characteristics of some signals (not exhaustive) tested on green sea turtles in the wild.

References

1. Technical Report Criteria and Thresholds for U.S. Navy Acoustic and Explosive Effects Analysis (Phase III) (2017).
2. Bürkner, P.-C. brms : An R Package for Bayesian Multilevel Models Using Stan. J. Stat. *Soft.* **80**. (2017).
3. Plummer, M., Best, N., Cowles, K., CODA: Convergence Diagnosis and Output Analysis for MCMC. *R News* **6**, 7–11 (2006).
4. R Core Team, R: A language and environment for statistical computing. https://www.R-project.org/ (2022).
